# Effects of a Multicomponent Exercise Program on Prevalence and Severity of the Frailty Syndrome in a Sample of Italian Community-Dwelling Older Adults

**DOI:** 10.3390/healthcare10050911

**Published:** 2022-05-13

**Authors:** Anna Mulasso, Mattia Roppolo, Alberto Rainoldi, Emanuela Rabaglietti

**Affiliations:** 1 Neuromuscular Function Research Group, Department of Medical Sciences, School of Exercise and Sport Sciences, University of Torino, 10143 Torino, Italy; alberto.rainoldi@unito.it; 2 Department of Psychology, University of Torino, 10124 Torino, Italy; mattia.roppolo@unito.it (M.R.); emanuela.rabaglietti@unito.it (E.R.)

**Keywords:** physical frailty, frailty prevention, physical exercise program, longitudinal study, Italian community-dwelling older adults, health promotion interventions

## Abstract

Background: Frailty is a well-known condition that leads to a lack of resilience, with a reduced homeostatic capacity and a consequent higher risk of suffering adverse health outcomes. This study investigated the effectiveness of an exercise program to improve and reverse physical frailty amongst Italian older adults. Methods: One hundred and twenty-three community dwelling older adults (mean age 74 years, SD = 6; 64% women) were involved in an experimental (EG; *n* = 62) and a control (CG; *n* = 61) group. Frailty was assessed at baseline and after the intervention using an adapted version of the frailty phenotype. The EG took part in a 16-week exercise program, consisting of endurance, strength, balance and flexibility exercises, while the CG maintained the same routine. Results: After the exercise program, the EG was more robust than the CG (F = 43.51, *p* < 0.001). Within the EG, 46% of pre-frail and 50% of frail people reached the robust and pre-frail levels, respectively. Effects of training were higher in frail and pre-frail people (reduction of frailty of 0.67 and 0.76 points, respectively) compared to robust ones (who frailty levels increased by 0.23 points; F = 11.32, *p* < 0.001). Conclusions: A multicomponent exercise program may be effective at improving and reverting frailty, specifically for frail and pre-frail people.

## 1. Introduction

Ever since the objective to live longer was accomplished by the Western world (in the last 100 years, life expectancy was increased by more than 30 years) [1], the new societal challenge has become to understand how to maximize the health status and quality of life in the later stages of life. One way to do so is to compress morbidity through specific interventions targeted to postpone the incidence of diseases which would assist in maintaining the physical, mental and social health status of individuals at a high level.

It is already well known that frailty is an important ageing-related indicator to measure health functions in old age. Frailty has been conceptualized as a loss of functional reserve, an inability to react to life events, an increased risks of adverse health outcomes, and an altered homeostatic capacity caused by a multiple and interactive complex system [2,3,4,5]. Gobbens et al. [6] defined frailty as “a dynamic state affecting an individual who experiences losses in one or more domains of human functioning”.

Frailty negatively influences physical functioning such as mobility, balance, muscle strength, and endurance [7,8], and increases the risk for fall incidence, disability, hospital admissions, institutionalization and mortality [2,9,10]. Frailty also worsens quality of life [11,12,13].

Strong evidence supports the role of regular physical exercise as a countermeasure for loss of physical functioning and clinical adverse outcomes in conditions of frailty [14,15]. Frail individuals can benefit from exercise programs, improving different outcome measures such as muscle strength [16,17,18,19,20], mobility [18,19,20], endurance [17], balance [17,21], fear of falling [19], incidence of falls [21], and functional ability [21]. Similarly, the same beneficial effects can be found in pre-frail individuals [22,23].

Moreover, physical exercise interventions can have a fundamental and direct role on the cycle of frailty, acting in the whole set of dysregulated mechanisms related to frailty and incidence of adverse health outcomes [1]. Nowadays, an increasing number of authors implemented multi-factorial and interdisciplinary programs (i.e., nutrition, physical exercise, psychosocial programs, etc.) for tackling frailty in frail [24,25,26], and pre-frail people [25,26,27,28,29]. Those types of interventions seem to be effective in preventing frailty. On the one hand, they take into account a variety plethora of activities and functions strictly related to the activities of daily living, acting on different domains of individual functioning; on the other hand, though, they are complex and challenging to perform by older adults. All these interventions include the physical exercise component, yet it is not possible to isolate the single contribution of exercise in frailty prevention.

Currently, studies that focus directly on the effects of exercise programs on physical frailty as an outcome measure are still scarce, as highlighted in recent reviews [30,31,32]. The study by Cesari et al. (2015) [33] reported the effectiveness of an exercise training which included aerobic, strength, flexibility and balance exercises for a 12-month period on frailty prevalence in a sample of community-dwelling older adults. Similarly, Arrieta et al. (2019) [34] reported on the beneficial effects of a six-month multicomponent exercise which reduced frailty levels in residents of long-term nursing homes. Ng et al. (2015) [25] also found that frailty was reduced amongst frail and pre-frail people when participating in a 24-week exercise program based on strength and balance, which was divided into 12 weeks of classes and 12 weeks at home. Furthermore, Tarazona-Santabalbina et al. (2016) [35] found that a multicomponent exercise program based on proprioception, aerobic, strength, and stretching exercises for a 24-week period (five days per week) had positive effects on frailty and physical functioning in a sample of frail people. Lastly, Losa-Reyna et al. (2019) [36] tested the effectiveness of a six-week concurrent power and high-intensity interval training to improve frailty in a small sample of frail people. All these studies used the frailty phenotype as a measure of frailty identification. In addition, it is also interesting to clarify whether the effects of exercise programs may differ according to the severity of frailty (robust, pre-frail and frail people). Thus far, only the study of Takano et al. (2017) [37] has found that the effects of interventions may be similar for pre-frail and robust older adults. Taking into consideration the single frailty criteria, physical exercise seems to generate improvements for each of them, and specifically in the areas of weakness [25,38,39], slowness [25,39], low physical activity [33,39], and poor endurance and energy [39]. Given the different characteristics of exercise programs and the heterogeneous study populations, many uncertainties exist with regard to frailty reversion and/or reduction.

Therefore, this study aims to evaluate the effects of a physical exercise program on the prevalence and severity of the physical frailty syndrome in a group of Italian community-dwelling older adults. Specifically, we investigate if the exercise program is able to: (i) reverse pre-frailty and frailty conditions to a less severe frailty status by identifying frailty criteria that is more receptive to the intervention; and (ii) improve frailty syndrome by analyzing differences according to frailty levels (robust, pre-frail and frail participants).

## 2. Materials and Methods

### 2.1. Study Population and Procedures

The sample size for this study was based on a 95% power to detect a minimum effect size (f) of 0.405 in an ANOVA (fixed effects, one-way) for three groups [40]. The alpha error probability was fixed at 5% and the statistical power (1-beta error probability) at 95%. This required a total sample of 99 participants. Additional participants were calculated to allow for the effect of dropout on post-test, and additional participants were computed. A sample of 135 older adults were enrolled in the study.

Participants were recruited from a list prepared by general medical practitioners. The inclusion criteria were the following: (i) they were aged 65 years and older, (ii) they had not regularly participated in moderate or vigorous physical exercise in the past year, (iii) they could walk independently (people using a walker were eligible), and (iv) they had a Mini-Mental State Examination (MMSE) of 25 or higher, indicating absence of cognitive impairment [41].

Participants were excluded if they had severe health problems that contraindicated physical training. Individuals who had previously participated in other studies were excluded. All participants in the study lived in the region of Piedmont (NW Italy) and were retired. No rewards nor incentives were offered to participate in the study. The Ethical Committee of the University of Torino approved the study protocol. In accordance with Italian law and the ethical code of the American Psychological Association (2002) [42], each participant provided written informed consent.

This was a longitudinal study conducted from 2015 to 2016. Data were collected at baseline (T0) and four months later, immediately after the end of the exercise program (T1). Tests were always administered in the same order and individually to each participant by trained staff consisting of a psychologist and an expert in physical exercise for older adults. The psychologist administered the cognitive test and collected socio-demographic variables. The expert in physical exercise administered the physical testing.

**Figure 1:**
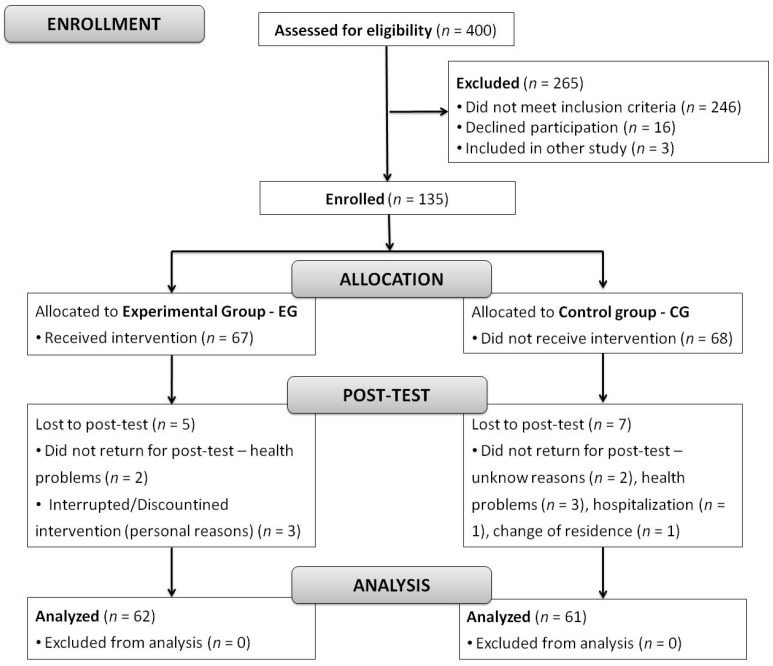
Consort diagram.

Following the baseline evaluation, participants were assigned to the experimental (EG; *n* = 67) or the control group (CG; *n* = 68) and matched in pairs by using sampling procedures which considered variables of interest such as gender, age, level of education, physical frailty status, presence of chronic diseases, etc. (Figure 1). The EG took part in a 16-week physical exercise program, while the CG maintained the same routine (e.g., they did not engage in regular exercise, performing only the usual activities they did prior to enrollment). Except for the exercise program, the participants of both groups were treated similarly.

A total of 5 participants (7.5%) dropped out from the EG and 7 (10.3%) from the CG. The reasons for dropping out from the program were not connected to study participation. No differences were found for demographic and individual characteristics, cognitive functions and physical frailty between participants who completed the study and those who dropped out.

### 2.2. Intervention

The exercise program lasted 16 weeks and consisted in a twice a week intervention of 70 min for each session on non-consecutive days. The exercise program was performed in groups composed of 12–15 participants (participants were not divided according to the severity of their frailty). The exercise program was led by one trainer, with a degree in physical and sports education and a specialization in adapted physical activity for older adults. The trainer had previous work experience in the field of exercise for older adults and had sufficient training (e.g., didactic sessions, ongoing training) to deliver the exercise program at a high level. Based on exercise recommendations for older adults [43,44], every session consisted of mixed activities which included endurance/walking exercises, resistance training, balance training, range of movement, breathing and stretching exercises. The program was designed so that the first weekly session was mostly dedicated to balance and resistance training, while the second session was primarily focused on endurance and walking. Training was mainly based on exercises and movements similar to daily tasks (e.g., chair rise, carrying shopping bags, climbing stairs, etc.) to foster autonomy. The equipment used during the exercise program was composed of resistance bands, hand weights, steps, small balls, sponge and tennis balls, etc. The characteristics of the exercise program are detailed in Table 1.

During the exercise program, stimuli evolution and progression were considered to increase physical fitness and physiological response to the training. Perceived effort was monitored using the modified Borg Rating of Perceived Exertion scale. Participants were asked to perform endurance exercises with a gradual increase in intensity each month (i.e., 3–4 “moderate”–“somewhat hard” in the first month, and 6–7 “hard” in the last month). Resistance exercises were initially performed at 25% of 1 repetition maximum (i.e., 2 sets of 30 repetitions) to 70% in the last month (4–5 sets of 10–12 repetitions). For balance, range of movement, breathing and stretching exercises, progression was established on the basis of movement quality. During each session, the trainer recorded data related to intensity, volume and quality of the exercises, and any adverse events related to each participant. During the session, the trainer constantly monitored participants’ physiological parameters such as heart rate, skin color, etc. After each session, the trainer would follow up by asking participants questions regarding their level of confidence in the acquisition of the trained skills.

### 2.3. Measures

Physical frailty was measured by using an adapted version of the frailty phenotype of Fried et al. (2001) [2], similar to those already tested in a previous study [45], and which consists of five indicators:

1. Shrinking was measured as a body mass index (BMI) less than 21 kg/m^2^ [46]. Weight was detected by a Tanita Body Composition Analyzer BF-350, and height by an anthropometer. Weight and height were collected with an accuracy of 0.1 kg and 0.01 m, respectively.
2. Weakness was evaluated by handgrip strength using the Smedley hand dynamometer (baseline 12-0286). We administered three trials for each hand with a pause of about 20 s between trials (always alternating right and left hands). The higher mean values between right and left hands were used for analysis. The same cut-off scores of Cardiovascular Health Study were applied to identify frail individuals for this criterion [2].
3. Poor endurance and energy were assessed by using the following two items of the Center of Epidemiologic Studies Depression scale (CES-D) [47]: (a) “I felt that everything I did was an effort”, (b) “I could not get going”. The statements referred to the previous week. Those who answered “a moderate amount of the time (3–4 days)” or “most of the time” to at least one of the questions resulted in a positive result for the endurance and energy component.
4. Slowness was evaluated by using the Timed Up and Go test (TUG) [48]. At the command “GO”, participants were asked to stand up from a chair, walk 3 m, turn around a cone, walk back to their chair and sit down at their usual pace. The test was performed once. A TUG cut-off of 10 s or more was used to categorize participants level of frailty based on their slowness [49,50].
5. Low physical activity was identified in participants who did not engage in leisure activities such as hiking, gardening, dancing or cycling at least once a week, regardless of their participation in the exercise program proposed in this study [46].

Participants were categorized as frail if they met three or more criteria, pre-frail if they met one or two of these criteria, and robust if they met none of the criteria [2].

Cognitive impairment was evaluated using the Mini Mental State Examination [41]. It was administered as a screening tool adopting a cut-off of 25 points or more. Lastly, age, gender, level of education, and previous work experience were self-reported information. Three questions about health status were asked: (1) “Do you usually take medication? Yes/No”, considering exclusively medicines taken on a regular basis (vitamins and dietary supplements were not taken into account); (2) “How do you rate your own health? Poor/Fair/Good/ Excellent”; (3) “Do you have one or more chronic diseases? Yes/No”.

### 2.4. Statistical Analysis

Statistical analyses were conducted using the Statistical Package for Social Sciences (SPSS), version 26.0 (SPSS Inc., Chicago, IL, USA). Statistical significance level was fixed at alpha < 0.05 for all tests.

Descriptive statistics were first conducted to describe all the study’s variables and to investigate the transition rate from a higher level of frailty level to a lower level. A t-test for the unpaired sample or chi-square test were executed to identify any differences at baseline between the EC and the CG for socio-demographic and individual characteristics, cognitive functions, and physical frailty.

Second, to test the effects of physical training on frailty, a one-way analysis of covariance (ANCOVA) was carried out, using participant’s age, gender, presence of chronic diseases, cognitive functions, pharmacotherapy, perception of health and value of physical frailty at baseline as covariates. Furthermore, the change in physical frailty between post-test and pre-test was calculated. While controlling for participant’s age, gender, presence of chronic diseases, cognitive functions, pharmacotherapy and perception of health, the ANCOVA test, together with the Sidak post-hoc test, were used to test differences of change in physical frailty among sub-groups of participants (robust, pre-frail and frail people) pertaining to the EG and the CG.

## 3. Results

### 3.1. Baseline Participants’ Characteristics

Table 2 shows baseline characteristics of the study’s participants and compares the EG and the CG for variables of interest. The whole sample had a mean age of 74 years (range 65–90, SD = 6). Most of the participants were female (*n* = 79, 64.2%), had a level of education corresponding to primary school (*n* = 61, 49.6%), were married or cohabiting (*n* = 66, 53.7%), and performed manual work before retirement such as farmer, mason, worker, waiter/waitress, etc. (*n* = 64, 52.0%). A large number of participants reported taking medicines (*n* = 107, 87.0%), and to have a fair (*n* = 50, 40.7%) or good (*n* = 68, 55.3%) health status. More than half of the participants (*n* = 66, 53.7%) reported having at least one chronic condition. The mean score obtained at the MMSE was 28.5 (SD = 1.9) points, confirming the high level of cognitive functioning of the participants. According to Fried’s operational definition, 58.5% (*n* = 72) were classified as pre-frail, 22.0% (*n* = 27) as robust, and 19.5% (*n* = 19) as frail. Overall, the mean score of physical frailty was 1.44 (SD = 1.12). Fried’s criteria with higher prevalence were “poor endurance and energy”, “weakness” and “low physical activity level” with 46.3%, 40.7% and 31.7%, respectively.

No differences were found in socio-demographic and individual characteristics, cognitive functions, and physical frailty between EG and CG at baseline. A slight imbalance was found for gender distribution (*p* = 0.070), with a higher number of women in the CG compared to the EG. Regarding physical frailty, there was a higher, but not statistically significant, prevalence of individuals who had a result of frail for “poor endurance and energy” in the EG compared to the CG (*p* = 0.057); on the contrary, there were more frail people for “low physical activity level” in the CG compared to EG (*p* = 0.071). Prevalence data for “shrinking”, “weakness” and “slowness” did not show statistically significant differences between the EG and the CG.

### 3.2. Drop-Out and Adherence

In total, 123 participants (91.1%) completed the study. Participants did not experience physical or medical problems during the exercise program; all the training sessions were completed. The mean adherence to the exercise program was 82% corresponding to 26/32 sessions (range 72–100%). The adherence to the program was not statistically different (*p* = 0.096) among robust (78%), pre-frail (83%) and frail (81%) participants.

### 3.3. Effects of Training

Considering the levels of physical frailty (robust, pre-frail and frail), 58% (*n* = 36) of the EG did not change their degree of physical frailty between T0 and T1 (77% of robust, 54% of pre-frail and 50% of frail). Among robust individuals, 23% (*n* = 3) worsened their frailty level at T1, passing to a pre-frailty level; while 46% (*n* = 17) of pre-frail participants resulted robust at T1, and 50% (*n* = 6) of those who were frail reached the pre-frailty level. On the contrary, a total of 77% (*n* = 47) of the CG maintained the same frailty level they had at baseline; in particular, 50% (*n* = 7) of those classified as robust, 86% (*n* = 30) of pre-frail and 83% (*n* = 10) of frail. Half of the robust people at baseline (*n* = 7) were shown to be pre-frail at T1, and 14% (*n* = 5) of those classified as pre-frail became frail, indicating a worsening of their frailty level; instead, 17% (*n* = 2) of frail individuals reached the pre-frailty level at T1.

With regards to the analysis of each frailty criterion, the major changes between T0 and T1 were registered for “poor endurance and energy” (EG: 5 people who became frail vs. 23 people who became robust at T1; CG: 15 people resulted frail vs. 7 people resulted robust at T1), “weakness” (EG: 2 people who worsened vs. 10 people who improved their condition at T1; CG: 15 people who worsened vs. 8 people who improved at T1), and “slowness” (EG: 2 people resulted frail vs. 5 people resulted robust at T1; CG: 11 people who worsened vs. 6 people who improved at T1). Limited changes between T0 and T1 occurred for “shrinking” and “low physical activity” in both the EG and the CG (Table 3).

After controlling for participant’s age, gender, presence of chronic diseases, cognitive function, pharmacotherapy, perception of health and value of physical frailty at baseline, the one way ANCOVA showed a significant difference in post-test for physical frailty between the EG and the CG, F(1114) = 43.51, *p* < 0.001, η^2^_p_ = 0.276. The adjusted R square of the model was 62.4%. Specifically, the EG improved its level of physical frailty between T0 and T1 (with a score of 1.48 at T0 and 0.95 at T1), while the CG worsened (from a score of 1.39 at T0 to 1.74 at T1) (Table 4).

Within the EG, changes in physical frailty were statistically significant among robust, pre-frail and frail individuals, F(2, 53) = 11.32; *p* < 0.001, η^2^_p_ = 0.299, controlling for participant’s age, gender, presence of chronic diseases, cognitive function, pharmacotherapy and perception of health. The model explained 26.3% of the variance. Specifically, robust individuals became more frail between T0 and T1, passing from 0 to 0.23 points. Pre-frail and frail groups improved their frailty condition, with a frailty reduction of 0.76 and 0.67 points, respectively. Significant differences were found between: (i) robust and frail (*p* = 0.002), and (ii) robust and pre-frail individuals (*p* < 0.001).

On the contrary, the CG did not show significant differences among the three frailty groups for changes in physical frailty. In general, all the frailty groups presented a slight worsening of their frailty condition between T0 and T1, with an increase of frailty severity of 0.57, 0.34, and 0.08 points, respectively, for robust, pre-frail and frail individuals (Table 5).

## 4. Discussion

This study investigated if older adults who participated in a 16-week exercise program could have beneficial effects which reversed and improved their frailty condition in comparison to a CG. The study contributes to knowledge about the rate of frailty and pre-frailty reversion and the effectiveness of an exercise program at different levels of frailty. Specifically, we analyzed changes of physical frailty among robust, pre-frail and frail people between pre- and post-test.

First, our results showed that the participants in the EG were significantly less frail after the intervention compared to the CG participants, supporting the role of an exercise program in addressing physical frailty. In particular, we found that almost half of pre-frail people restored to a complete condition of robustness; and half of frail individuals reached a pre-frailty level after the intervention (none of them regained robustness). On the contrary, in the CG, there were no pre-frail individuals who regained robustness, and a very limited number of frail individuals who returned to a pre-frailty level. Consistent with our results, Cesari et al. (2015) [33] found a reduction of frailty prevalence after a 12-month exercise program in a sample of community-dwelling older adults (prevalence of 10.0% for the intervention group compared to 19.1% for the control group). Instead, the study of Tarazona-Santabalbina et al. (2016) [35] tested the effectiveness of physical exercise on frail older adults, and Ng et al. (2015) [25] on pre-frail and frail participants. The last two studies reported a reversion of frailty in 31.4% and 41.3% of the cases, respectively. Consistent with previous findings, our study demonstrated that pre-frailty and frailty can be considered reversible conditions prevented by an exercise program. However, it is important to understand through further research if frailty can be only reversed to pre-frailty, as observed in our study, or if frailty can be completely reversed to robustness. If we consider frailty as a precursor state of disability and of negative outcomes such as falls, loss of quality of life, institutionalization, hospitalization, death, etc., preventive strategies which reduce or postpone frailty are urgently needed, as they allow improvement of individuals’ health and quality of life, as well as savings in healthcare costs.

Furthermore, whenever possible, delivery of the exercise programs in groups is suggested, as we did in our study, since the effectiveness for preventing frailty may be higher [51].

Consistent with other similar studies (including Ng et al., 2015) [25], the lower rate of drop out (especially for the EG) also supports the feasibility of implementing physical exercise in people who are frail or at risk of becoming frail. Second, it is interesting to note the different effects this exercise program had on robust, pre-frail and frail people. In fact, after the exercise program, frail and pre-frail groups improved their frailty condition, while the robust people slightly worsened. Consequently, physical training seems to be more effective in frail and pre-frail individuals when compared to robust individuals. This finding suggests that the intensity of the intervention and the difficulty of the tasks proposed in this study were probably underestimated for the most fit people and, consequently, it is necessary to differentiate the quantity, intensity, and quality of the exercise stimuli so that the exercise program is effective for all participants according to their frailty level. In this regard, it may be possible in future studies to increase the perceived effort and heart rate intensity during the endurance and resistance phases of the intervention and to design more complex training sessions for robust individuals. The use of other measures (e.g., multidimensional frailty tools) that can better classify robust participants should also be considered to implement a more specific exercise program for robust individuals.

It should be noted that, in general, more compromised people (i.e., frail and pre-frail subjects) have the greatest gain in comparison to fit individuals (i.e., robust subjects) [33,52]. In fact, frail and pre-frail older adults have lower levels of functional abilities and, as a consequence, a better chance to increase them [53]. In contrast, the study of Gill et al. (2002) [54] reported, after a physical exercise program, less beneficial effects on disability for the frailest participants. This difference can probably be explained in terms of outcome analyzed (disability in the study of Gill et al. vs. physical frailty in the present paper). We can assume that in people with a higher risk profile, physical training acts more immediately on physical frailty as a direct effect, and it requires a longer period to generate an effect on disability. Disability has a complex nature and is determined by the interactions of various risk factors (i.e., frailty) [6,54].

Our results also show that the frailty condition worsened in all the CG subgroups (robust, pre-frail and frail people), with a greater worsening in robust and pre-frail individuals. It is clear that the substantial worsening of the frailty condition in the CG subgroups may be explained by the choice to include only people who were not used to exercising. An inactive lifestyle is a factor that exacerbates frailty, increasing the risk of transition to a more severe frailty status [55]. Robust individuals of the CG are the subgroup whose condition worsened more between pre and post-test, while the robust individuals of the EG showed a weak deterioration of their condition. Nevertheless, it is worth underlining that the negative trend of frailty observed in the robust participants of the EG is lower than it was in the robust participants of the CG. This means that the exercise program implemented in this study has the potential to slow down the progression of physical frailty in robust older adults.

Lastly, regarding the individual frailty criteria, our exercise program seems to generate more consistent improvement in weakness, as reported by Kwon et al. (2015) [38] in a sample of pre-frail women, and by Liao et al. (2019) [39] in a sample of pre-frail and frail older adults; and poor endurance and energy as determined by Liao et al. (2019) [39], followed by slowness. On the contrary, the CG experienced a worsening of their condition for these criteria; whereas, for the criteria of low physical activity, as well as shrinking, there were limited changes for both groups (EG and CG). These results can be explained considering the characteristics of the participants. The participation of individuals who do not usually exercise leads to a more consistent and faster improvement in physical fitness (i.e., acting directly on strength levels and consequently on the weakness criterion, and on the perception of fatigue linked to poor endurance and energy). It should also be noted that the criteria of weakness and poor endurance and energy already had a higher prevalence at baseline. Exercise programs can certainly have an impact on the low physical activity criteria, but a more comprehensive intervention is probably needed to generate a positive change, since increasing physical activity (without considering physical activity performed during training sessions, as in our study) is a process that requires awareness of the importance of adopting an active lifestyle.

From a practical point of view, the inclusion of older adults who were not accustomed to exercising, combined with the low dropout rate and high exercise adherence, and the implementation of multicomponent physical exercise based on movements similar to daily tasks with potential positive effects on people’s lives, were important strengths of the study. Considering the innovative aspects, our results support the idea that physical frailty can be improved and/or reversed by an exercise program by providing information on the criteria more susceptible to improvement. Therefore, these findings enrich the previous literature, which is still limited. The present results should also be interpreted in the context of some limitations. First, participants were recruited in a small area of Italy without using a stratifying sampling procedure, and with strict inclusion criteria, making it difficult to generalize the results to the entire Italian population of older adults. Furthermore, some subgroups of participants whose data were taken into account for the analysis (e.g., frail individuals) were composed of a limited number of individuals. Another limitation concerns the adoption of an adapted version of the frailty phenotype, making comparison with results from other studies difficult. Another possible limitation may have been the exercise group composition with people who had heterogeneous levels of frailty; this may have resulted in greater difficulties to adapt the training to their abilities. Finally, an additional limitation is related to the absence of follow-up in order to evaluate whether there were any trends of the effects of the physical exercise program over time.

Further analyses on larger samples of older adults throughout the whole national territory must be performed to increase validity and scientific evidence on the benefits of exercise training on physical frailty. Furthermore, larger studies need to be designed to test the maintenance of exercise effects over medium to long term periods. Finally, the effectiveness of an exercise program should be tested within a wider definition of frailty that includes the psychological and social domains in addition to the physical component of frailty.

Future studies should also focus on the implementation of multidomain interventions to prevent frailty, which currently seem to be more effective than physical exercise alone [25].

## 5. Conclusions

These results suggest the positive effect of a physical exercise program on physical frailty in a sample of older adults who were not used to exercising, demonstrating the possibility of improving and reverting frailty. Positive effects of intervention seem to be more evident in pre-frail and frail individuals. Taking into consideration the single frailty criteria, poor endurance and energy, and weakness, followed by slowness, seem to be more consistently affected by the exercise program when compared to other frailty criteria.

The feasibility and the effectiveness of implementing a multicomponent exercise training has major practical implications for the design of future program for older adults at risk of physical frailty. Specifically, a more targeted exercise program for robust individuals may be needed, with increased quantity and the creation of more complex exercise stimuli. Lastly, in order to positively influence each frailty criteria, the implementation of multidomain interventions (e.g., physical exercise in association with a dietary regime) should be considered.

## Figures and Tables

**Table 1:** Characteristics of the exercise program (day 1 and day 2).

Intervention Characteristics: 16 Weeks, 2 Sessions/Week, 70 Min/Session	
Day 1	Day 2
Warm up (10 min)	Warm up (5 min)
Balance training (15 min)	Endurance walking exercises (25 min)
Resistance training (20 min)	Balance training (15 min)
Walking exercises (15 min)	Resistance training (15 min)
Range of movement (10 min)	Breathing and stretching (10 min)

**Table 2:** Baseline characteristics for the whole sample, the experimental group (EG) and the control group (CG).

Variable	Whole Sample *n* = 123	EG *n* = 62	CG *n* = 61	*p* Value ^1^
Age, years, mean (SD), years	74 (6)	73 (5)	74 (6)	0.917
Gender, *n* (%) of female	79 (64.2)	35 (56.5)	44 (72.1)	0.070
Level of education, *n* (%)				0.796
Primary school, 5 years	61 (49.6)	28 (45.2)	33 (54.1)	
Secondary school, 8 years	38 (30.9)	21 (33.9)	17 (27.9)	
High school diploma, 13 years	20 (16.3)	11 (17.7)	9 (14.8)	
University degree, 18 years	4 (3.3)	2 (3.2)	2 (3.3)	
Marital status, *n* (%)				0.037
Married/Cohabiting	66 (53.7)	37 (59.7)	29 (47.5)	
Not married	5 (4.0)	3 (4.8)	2 (3.3)	
Widowed	45 (36.6)	16 (25.8)	29 (47.5)	
Divorced	7 (5.7)	6 (9.7)	1 (1.7)	
Past job, *n* (%)				0.451
Housewife	13 (10.6)	8 (12.9)	5 (8.2)	
Manual	64 (52.0)	29 (46.8)	35 (57.4)	
Non-manual	46 (37.4)	25 (40.3)	21 (34.4)	
Chronic disease (CD), *n* (%) of people with at least 1 CD	66 (53.7)	30 (48.4)	36 (59.0)	0.237
Pharmacotherapy, *n* (%) of Yes	107 (87.0)	54 (87.1)	53 (86.9)	0.972
Perception of health, *n* (%)				0.239
Poor	1 (0.8)	0 (0.0)	1 (1.6)	
Fair	50 (40.7)	21 (33.9)	29 (47.5)	
Good	68 (55.3)	38 (61.3)	30 (49.2)	
Excellent	4 (3.3)	3 (4.8)	1 (1.6)	
MMSE, mean (SD)	28.5 (1.9)	28.2 (1.9)	28.9 (1.9)	0.056
Physical frailty, mean (SD)	1.44 (1.12)	1.48 (1.10)	1.39 (1.14)	0.655
Physical frailty status, *n* (%) ^2^				0.959
Robust	27 (22.0)	13 (21.0)	14 (23.0)	
Pre-frail	72 (58.5)	37 (59.7)	35 (57.4)	
Frail	24 (19.5)	12 (19.4)	12 (19.7)	

^1^ Based on independent *t*-test for continuous variables and on chi-squared test for categorical variables. ^2^ According to the Cardiovascular Health Study criteria, individuals with three or more criteria were classified as frail, those with one or two criteria as pre-frail, and those without criteria as robust. Abbreviations: SD, standard deviation; BMI, body mass index; TUG, Timed Up and Go test; MMSE, Mini Mental State Examination. A higher score corresponded to a better cognitive status.

**Table 3:** Comparison of changes between EG and CG from baseline to post-test for each CHS frailty criterion.

CHS Frailty Criteria	Group	T0 Baseline *n* (%)	T1 Post-Test *n* (%)	
			No Frail	Frail
Shrinking	EG	No frail	58 (94)	2 (3)
		Frail	2 (3)	0 (0)
	CG	No frail	60 (98)	1 (2)
		Frail	0 (0)	0 (0)
Weakness	EG	No frail	34 (55)	2 (3)
		Frail	10 (16)	16 (26)
	CG	No frail	22 (36)	15 (25)
		Frail	8 (13)	16 (26)
Poor endurance and energy	EG	No frail	23 (37)	5 (8)
		Frail	23 (37)	11 (18)
	CG	No frail	23 (38)	15 (25)
		Frail	7 (11)	16 (26)
Slowness	EG	No frail	45 (73)	2 (3)
		Frail	5 (8)	10 (16)
	CG	No frail	36 (59)	11 (18)
		Frail	6 (10)	8 (13)
Low physical activity	EG	No frail	47 (76)	0 (0)
		Frail	4 (6)	11 (18)
	CG	No frail	37 (61)	0 (0)
		Frail	0 (0)	24 (39)

Data are presented as mean (%). Abbreviations: EG, experimental group; CG, control group.

**Table 4:** Effects of training on physical frailty based on one-way ANCOVA (*n* = 123).

	EG		CG				
	T0	T1	T0	T1	F(1114)	*p* Value	ƞ ^2^_p_
Physical frailty	1.48 (1.10)	0.95 (1.08)	1.39 (1.14)	1.74 (1.15)	43.51	<0.001	0.276

Data are presented as mean (SD). Participant’s age, gender, presence of chronic diseases, cognitive functions, pharmacotherapy, perception of health and value of physical frailty in baseline are used as covariates. Abbreviations: SD, standard deviation; EG, experimental group; CG, control group.

**Table 5:** Comparison of physical exercise effects on different levels of physical frailty in EG and CG based on one-way ANCOVA.

Group	Physical Frailty Status	*n*	Physical Frailty Change, Mean (SD)	F	*p*-Value	ƞ ^2^_p_
EG	Robust	13	0.23 (0.44) *^,^**	11.32	<0.001	0.299
	Pre-frail	37	−0.76 (0.72)			
	Frail	12	−0.67 (0.78)			
CG	Robust	14	0.57 (0.65)	1.62	n.s.	0.059
	Pre-frail	35	0.34 (0.91)			
	Frail	12	0.08 (0.79)			

Data are presented as mean (SD). * *p* < 0.05 vs. pre-frail; ** *p* < 0.05 vs, frail. Participant’s age, gender, presence of chronic diseases, cognitive functions, pharmacotherapy and perception of health are used as covariates; physical frailty change (negative and positive numbers mean an improvement and a worsening of frailty condition, respectively). Abbreviations: SD, standard deviation; EG, experimental group; CG, control group; n.s. not significant.

## Data Availability

The data presented in this study are available on request from the corresponding author.
